# Dispositional Gratitude Moderates the Association between Socioeconomic Status and Interleukin-6

**DOI:** 10.1038/s41598-018-37109-1

**Published:** 2019-01-28

**Authors:** Andree Hartanto, Sean T. H. Lee, Jose C. Yong

**Affiliations:** 1 Singapore Management University, Singapore, Singapore; 2 National University of Singapore, Singapore, Singapore

## Abstract

Socioeconomic disparities in health are prevalent and growing in importance as a concern among academics, policymakers, and the general public. However, psychological resources that can narrow such disparities have not been well-examined. The current study examined the moderating role of dispositional gratitude in the relationship between socioeconomic status (SES) and inflammation risk as an index of health. Participants consisted of 1,054 midlife adults from the biomarker project of the Midlife in the United States. Inflammation risk was measured by interleukin-6 biomarker and SES was operationalized by education attainment and income. We found that dispositional gratitude significantly moderated the relationships between SES and interleukin-6. Among individuals with low dispositional gratitude, higher SES was significantly associated with lower levels of interleukin-6. However, the association between SES and interleukin-6 was not significant among individuals with high dispositional gratitude. More importantly, the findings remained robust after controlling for demographic characteristics, health status, health behaviours, and personality traits. Our findings suggest that gratitude may serve as an important psychological resource in attenuating health-related risk from socioeconomic stressors.

## Introduction

With increasing inequality in both developed and developing countries across the world^1^, socioeconomic disparities in health has become one of the most important public concerns and received notable empirical attention among researchers^2–4^. A large number of studies have shown that lower socioeconomic status (SES) is associated with increased incidence of negative health-related outcomes such as diabetes^5^, cardiovascular diseases^6,7^, and asthma^8,9^. It has been posited that such adverse health outcomes faced by lower SES individuals could be attributable to their disproportionate exposure to stressors in daily life and compounded by their tendency to appraise such stressors negatively^10,11^. As such, an important endeavour for researchers is to identify psychological resources that can potentially buffer against such injurious effects^12^. In this paper, we examine gratitude as one such potential psychological resource. Can dispositional gratefulness fortify the mind, serve as a defensive buffer against stressors such as those brought upon by socioeconomic disparity, and in turn help attenuate the relationship between low SES and poorer physical health?

While gratitude at a state level refers to a positive emotion that is experienced when receiving benefits from an external source, gratitude at the dispositional trait level refers to a general orientation towards perceiving and appreciating the positives in life^13,14^. A generally grateful response to life circumstances where individuals interpret daily life experiences positively can lead to improved subjective well-being^15,16^. By shifting affective perceptions of daily life events from negative to positive, gratitude is incompatible with the “negative triad” of beliefs underlying depression, namely negative views about oneself, the world, and the future^17^. Gratitude is associated with hedonic wellbeing (i.e., “subjective wellbeing” as characterized by higher positive affect, lower negative affect, and life satisfaction) and eudaimonic wellbeing (i.e., “psychological wellbeing”, characterized by environmental mastery, personal autonomy, purpose in life, positive relations with others, and personal growth^18,19^), both of which are associated with reduced likelihood of depression^20,21^. Indeed, various studies have shown that gratitude is associated with greater levels of optimism^22^, psychological well-being^23^, and overall life satisfaction^24^.

In terms of physical health, grateful individuals fare significantly better than less grateful individuals in part due to their better psychological health, higher proclivity for healthy activities, and greater willingness to seek medical help^25^. People who express more gratitude experience greater hedonic wellbeing and eudaimonic wellbeing and are more likely to have improved immune function^26^. In addition, gratitude is associated with lower self-reported levels of loneliness which in turn predicted more favourable self-reported physical health symptoms^27^. Compared to individuals with lower levels of dispositional gratitude, individuals with higher levels of dispositional gratitude are more oriented toward positive features of the environment and are thus more likely to seek out and foster personally and socially productive behaviours, including strengthening social bonds and engaging in healthy activities such as exercising more and adopting a healthier diet^14,21,27–29^.

Importantly, gratitude influences the way individuals approach and cope psychologically with daily life challenges and stressors. The overall positive outlook on life held by grateful people has been argued to increase their willingness to actively tackle and resolve problems as opposed to exhibiting behaviours characteristic of disengagement, withdrawal, or avoidance^30^. For instance, a study on caregivers of persons with dementia showed that dispositional gratitude predicted caregivers’ adoption of mental coping strategies such as positive reframing, acceptance, humour, emotional social support seeking, and religious coping, which in turn predicted lower levels of perceived caregiving burden and depressive symptoms^31^. Taking into account grateful individuals’ proclivity to adopt adaptive coping strategies when faced with stressors, we therefore postulate that high levels of dispositional gratitude enables low SES individuals to psychologically manage the heightened amount of daily life stressors that they may face, thereby buffering the negative impact of such stressors on health.

Studies examining the relationship between gratitude and physical health have mostly relied on self-report measures^25,27^. However, the use of self-report measures of physical health is especially problematic in studies of gratitude as grateful individuals may be subject to positivity bias and skewed physical health perceptions^32,33^. To date, only a few studies have extended their examination of the relationship between gratitude and physical health to include objective health measures such as inflammatory biomarkers. One of the few studies that employed objective health measures found that dispositional gratitude was related to a reduction in inflammatory biomarkers among 186 older adults^34^. Moreover, a study on gratitude journaling in 70 stage B heart failure patients found that writing regularly about the things one is grateful and appreciative for over a period of eight weeks significantly reduced participants’ inflammatory biomarker index and increased their parasympathetic heart rate variability response, which collectively indicates a reduction in risk of heart failure^35^. These findings may not yet be conclusive due to their small sample sizes, but the effective replication of findings supporting a relationship between gratitude and inflammation biomarkers has important implications because inflammation has also been implicated in the etiology of a wide range of diseases ranging from rheumatological diseases to osteoporosis and even Alzheimer’s disease^36^, in addition to playing a key role in cardiovascular disease pathology^37,38^.

Among the pool of commonly assessed inflammatory biomarkers which includes interleukin-6, C-reactive protein (CRP), and fibrinogen, interleukin-6 is the most pivotal in the context of SES. One study found that while all three biomarkers were inversely related to SES (in terms of education and income levels), interleukin-6 fully mediated the relationship between SES and the other two biomarkers^39^. This is attributable, at least in part, to the fact that hepatocyte production of both CRP and fibrinogen are strongly driven by interleukin-6 levels^40–43^. Hence, the association between SES and serum levels of CRP and fibrinogen may stem more fundamentally from the relationship between SES and serum levels of interleukin-6^39^. As such, studies examining the impact of SES on physical health often focus primarily on interleukin-6^44,45^. Consistent with the notion that lower levels of SES are associated with greater exposure to daily stressors and poorer physical health^10,11^, across a wide range of SES indicators such as income and education, lower levels of SES have been consistently related to higher levels of serum-circulating interleukin-6^45–48^.

Taken together, the aims of this present study are twofold. First, we aim to examine the moderating role of dispositional gratitude in the established relationship between SES and interleukin-6 with large samples (*n* = 1,054). Here, we hypothesized that socioeconomic disparities in interleukin-6 would be less prevalent among individuals with high gratitude than individuals with low gratitude. Next, we also aim to conceptually replicate whether dispositional gratitude is positively associated with interleukin-6 as the inflammation biomarker. Based on previous findings^34,35^, we hypothesized that dispositional gratitude would predict lower levels of interleukin-6—indicating lower inflammation risk—even after controlling for demographics, health status, health behaviours, and personality traits. The findings from our study will contribute importantly to informing future interventions aimed at attenuating the deleterious effects of low SES on physical health. In particular, the results from this present study would provide some indication of the viability of interventions aimed at fostering gratitude^35,49^ in curbing negative health outcomes associated with low SES.

## Results

### Education attainment

Before our moderation analyses, we first examined the direct relationship between education attainment and interleukin-6 in an unadjusted model. We found that education attainment was significantly, negatively associated with interleukin-6 levels (*B* = −0.03, *SE* = 0.009, *t* = −2.872, *p* = 0.004). Subsequently, we conducted moderation analyses on education attainment and dispositional gratitude. Our results are summarized in Table 1. In line with our prediction, we consistently observed significant two-way interactions of education attainment × gratitude on interleukin-6 across the four separate models, specifically in Model 1 when the model was unadjusted (*B* = 0.20, *SE* = 0.08, 95% CI = [0.050, 0.357], *t* = 2.61, *p* = 0.009), Model 2 when demographic variables were controlled (*B* = 0.20, *SE* = 0.08, 95% CI = [0.05, 0.35], *t* = 2.65, *p* = 0.008), Model 3 when we included health status and health-related behaviours as covariates (*B* = 0.19, *SE* = 0.08, 95% CI = [0.04, 0.33], *t* = 2.55, *p* = 0.011), and Model 4 when personality factors were controlled (*B* = 0.18, *SE* = 0.07, 95% CI = [0.04, 0.33], *t* = 2.43, *p* = 0.015).

**Table 1:** Model Summaries of Interleukin-6 with Education Attainment and Dispositional Gratitude as Predictors.

	Model 1		Model 2		Model 3		Model 4	
	Beta	*B* (*SE*)	Beta	*B* (*SE*)	Beta	*B* (*SE*)	Beta	*B* (*SE*)
**Main effect**								
Education Attainment	−0.09	−0.03 (0.01)**	−0.07	−0.02 (0.01)*	−0.05	−0.01 (0.01)	−0.04	−0.01 (0.01)
Gratitude	0.00	0.02 (0.19)	−0.01	−0.05 (0.19)	0.00	0.02 (0.19)	0.00	0.02 (0.20)
**Two-way interaction**								
Education Attainment × Gratitude	0.08	0.20 (0.08)**	0.08	0.20 (0.08)**	0.08	0.19 (0.07)*	0.07	0.18 (0.07)*
**Covariates**								
Age			0.24	0.02 (0.00)***	0.18	0.01 (0.00)***	0.16	0.01 (0.00)***
Gender			−0.02	−0.03 (0.05)	0.01	0.02 (0.05)	0.00	0.01 (0.05)
Marital status			−0.06	−0.10 (0.05)*	−0.05	−0.08 (0.05)	−0.06	−0.09 (0.05)
Citizenship			0.00	0.00 (0.35)	−0.00	−0.02 (0.34)	−0.00	−0.05 (0.34)
Number of chronic disease					0.18	0.04 (0.01)***	0.19	0.05 (0.01)****
Former smoker					0.02	0.04 (0.05)	0.03	0.04 (0.05)
Current smoker					0.06	0.13 (0.07)	0.06	0.14 (0.07)*
Alcohol					−0.06	−0.03 (0.01)*	−0.05	−0.03 (0.01)
Openness to experience							−0.07	−0.09 (0.05)
Conscientiousness							−0.09	0.14 (0.05)**
Extraversion							0.00	0.00 (0.05)
Agreeableness							0.03	0.05 (0.05)
Neuroticism							−0.08	−0.09 (0.04)*

*Note*: Gender was dummy coded with female as reference. Marital status was dummy coded with unmarried as reference. Citizenship was dummy coded with non-U.S. citizen as reference. Former and current smoker were dummy coded with non-smoker as reference. *p < 0.05, **p < 0.01, ***p < 0.001.

**Figure 1:**
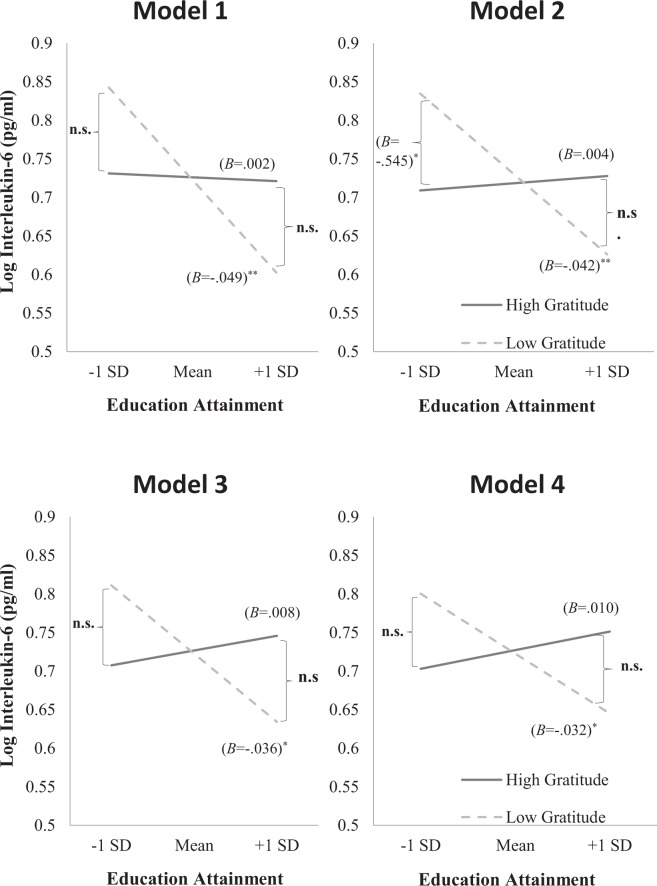
Simple slopes (i.e., unstandardized coefficients) of education attainment predicted interleukin-6 when trait gratitude was 1 *SD* above and below the mean across the four models (adjusted and unadjusted models). n.s. = not significant. *p < 0.05, **p < 0.01.

When we performed simple slopes analyses to probe these interactions (Fig. 1), we found that among participants with low dispositional gratitude, education attainment was negatively associated with interleukin-6 across the four separate models; Model 1 (*B* = −0.05, *SE* = 0.01, *t* = −3.87, *p* < 0.001), Model 2 (*B* = −0.04, *SE* = 0.01, *t* = 3.45, *p* < 0.001), Model 3 (*B* = −0.04, *SE* = 0.01, *t* = −2.94, *p* = 0.003), and Model 4 (*B* = −0.03, *SE* = 0.01, *t* = −2.54, *p* = 0.011). In contrast, among participants with high dispositional gratitude, education attainment was not significantly associated with interleukin-6 across the four separate models; Model 1 (*B* = 0.00, *SE* = 0.01, *t* = −0.16, *p* = 0.874), Model 2 (*B* = 0.00, *SE* = 0.01, *t* = 0.30, *p* = 0.761), Model 3 (*B* = 0.01, *SE* = 0.01, *t* = 0.53, *p* = 0.532), and Model 4 (*B* = 0.01, *SE* = 0.01, *t* = 0.78, *p* = 0.434). The results suggest that among individuals with low gratitude, those with higher education attainment have lower levels of interleukin-6 than those with lower education attainment even when demographic variables, health status, health behaviours, and personality traits were controlled. However, among individuals with high gratitude, those with higher education attainment have similar levels of interleukin-6 as those with lower education attainment.

It is noteworthy that, contrary to our prediction, there was no main effect of gratitude on interleukin-6 in all of our models; Model 1 (*B* = 0.02, *SE* = 0.19, 95% CI = [−0.362, 0.393], *t* = 0.08, *p* = 0.935), Model 2 (*B* = −0.05, *SE* = 0.19, 95% CI = [−0.428, 0.323], *t* = −0.27, *p* = 0.785), Model 3 (*B* = 0.02, *SE* = 0.19, 95% CI = [−0.354, 0.389], *t* = 0.09, *p* = 0.927), and Model 4 (*B* = 0.02, *SE* = 0.20, 95% CI = [−0.375, 0.414], *t* = 0.10, *p* = 0.923). Also, when taking into account education attainment, gratitude only emerged as a significant predictor among individuals with education attainment of one standard deviation above the mean in Model 2 (*B* = −0.54, *SE* = 0.26, 95% CI = [−1.060, −0.031], *t* = 2.08, *p* = 0.038).

### Income

Similar with education attainment, we first examined the direct relationship between income and interleukin-6 in the unadjusted model. We also found that income was significantly, negatively associated with interleukin-6 levels (*B* = −0.06, *SE* = 0.020, *t* = −3.052, *p* = 0.002). Our subsequent moderation analyses for income and dispositional gratitude are summarized in Table 2. Similar with the patterns found for education attainment, we also observed significant two-way interactions of income × dispositional gratitude on interleukin-6 across the four separate models, specifically in Model 1 when the model was unadjusted (*B* = 0.51, *SE* = 0.17, 95% CI = [0.17, 0.85], *t* = 2.95, *p* = 0.003), Model 2 when we included demographic variables as covariates (*B* = 0.48, *SE* = 0.17, 95% CI = [0.15, 0.81], *t* = 2.82, *p* = 0.005), Model 3 when we controlled for health status and health-related behaviours (*B* = 0.45, *SE* = 0.17, 95% CI = [0.12, 0.77], *t* = 2.69, *p* = 0.007), and Model 4 when personality factors were controlled (*B* = 0.40, *SE* = 0.17, 95% CI = [0.08, 0.73], *t* = 2.42, *p* = 0.016).

**Table 2:** Model Summaries of Interleukin-6 with Income and Dispositional Gratitude as Predictors.

	Model 1		Model 2		Model 3		Model 4	
	Beta	*B* (*SE*)	Beta	*B* (*SE*)	Beta	*B* (*SE*)	Beta	*B* (*SE*)
**Main effect**								
Income	−0.09	−0.06 (0.02)**	−0.07	−0.03 (0.02)	−0.05	−0.02 (0.02)	−0.04	−0.01 (0.02)
Gratitude	0.00	0.01 (0.19)	−0.01	−0.06 (0.19)	0.00	0.01 (0.19)	0.00	0.01 (0.20)
**Two-way interaction**								
Income × Gratitude	0.08	0.51 (0.17)**	0.08	0.48 (0.17)**	0.08	0.45 (0.17)***	0.07	0.40 (0.17)*
**Covariates**								
Age			0.24	0.02 (0.00)***	0.18	0.01 (0.00)***	0.16	0.01 (0.00)***
Gender			−0.02	−0.17 (0.05)	0.01	0.02 (0.05)	0.00	0.01 (0.05)
Marital status			−0.06	−0.90 (0.05)	−0.05	−0.07 (0.05)	−0.06	−0.08 (0.05)
Citizenship			0.00	0.03 (0.35)	−0.00	−0.09 (0.34)	−0.00	−0.04 (0.34)
Number of chronic disease					0.18	0.04 (0.01)***	0.19	0.05 (0.01)***
Former smoker					0.02	0.04 (0.05)	0.03	0.04 (0.05)
Current smoker					0.06	0.15 (0.07)*	0.06	0.15 (0.07)*
Alcohol					−0.06	−0.03 (0.01)*	−0.05	−0.03 (0.01)
Openness to experience							−0.07	−0.11 (0.05)*
Conscientiousness							−0.09	0.13 (0.05)**
Extraversion							0.00	0.00 (0.05)
Agreeableness							0.03	0.06 (0.05)
Neuroticism							−0.08	−0.09 (0.04)*

*Note*: Gender was dummy coded with female as reference. Marital status was dummy coded with unmarried as reference. Citizenship was dummy coded with non-U.S. citizen as reference. Former and current smoker were dummy coded with non-smoker as reference. *p < 0.05, **p < 0.01, ***p < 0.001.

**Figure 2:**
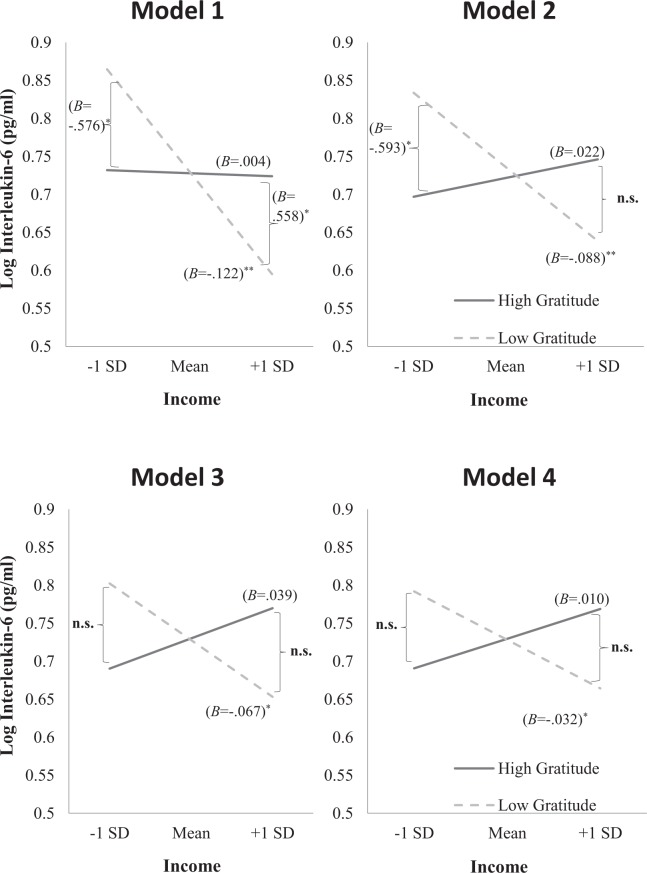
Simple slopes (i.e., unstandardized coefficients) of income predicted interleukin-6 when trait gratitude was 1 *SD* above and below the mean across the four models (adjusted and unadjusted models). n.s. = not significant. *p < 0.05, **p < 0.01.

Similarly, when we performed simple slopes analyses to probe the income × dispositional gratitude interactions (Fig. 2), we observed that among participants with low dispositional gratitude, income was negatively associated with interleukin-6 across four separate models; Model 1 (*B* = −0.12, *SE* = 0.02, *t* = −4.25, *p* < 0.001), Model 2 (*B* = −0.09, *SE* = 0.03, *t* = 3.03, *p* < 0.001), Model 3 (*B* = −0.07, *SE* = 0.03, *t* = −2.34, *p* = 0.020), and Model 4 (*B* = −0.06, *SE* = 0.03, *t* = −1.99, *p* = 0.047). In contrast, among participants with high dispositional gratitude, education attainment was not significantly associated with interleukin-6 across four separate models; Model 1 (*B* = 0.00, *SE* = 0.03, *t* = −0.13, *p* = 0.897), Model 2 (*B* = 0.02, *SE* = 0.03, *t* = 0.80, *p* = 0.425), Model 3 (*B* = 0.04, *SE* = 0.03, *t* = 1.30, *p* = 0.194), and Model 4 (*B* = 0.04, *SE* = 0.03, *t* = 1.28, *p* = 0.199). Taken together, the results suggest that among individuals with low gratitude, those with higher income have lower levels of interleukin-6 than those with lower income regardless of their demographic factors, health status, health behaviours, and personality traits. However, among individuals with high gratitude, those with higher income have similar levels of interleukin-6 as those with lower income.

It is also noteworthy that gratitude was not a significant predictor of interleukin-6 in all of our models; Model 1 (*B* = 0.01, *SE* = 0.19, 95% CI = [−0.385, 0.368], *t* = 0.04, *p* = 0.965), Model 2 (*B* = 0.06, *SE* = 0.19, 95% CI = [−0.439, 0.311], *t* = 0.33, *p* = 0.738), Model 3 (*B* = 0.01, *SE* = 0.19, 95% CI = [−0.362, 0.381], *t* = 0.05, *p* = 0.959), and Model 4 (*B* = 0.01, *SE* = 0.20, 95% CI = [−0.386, 0.400], *t* = 0.04, *p* = 0.971). When taking into account level of income, gratitude was negatively associated with interleukin-6 among individuals with lower income in Model 1 (*B* = −0.58, *SE* = 0.26, 95% CI = [−1.092, −0.060], *t* = 2.19, *p* = 0.029) and Model 2 (*B* = −0.59, *SE* = 0.26, 95% CI = [−1.104, −0.083], *t* = 2.28, *p* = 0.023). Interestingly, we also observed that gratitude was positively associated with interleukin-6 among individuals with higher income in Model 1 (*B* = −0.56, *SE* = 0.28, 95% CI = [0.010, 1.108], *t* = 2.00, *p* = 0.046). However, this result should be interpreted with caution as the significant positive association was not significant in the other models where confounding variables were controlled for.

## Discussion

In the current study of a large sample of 1,054 middle-aged and older adults, we consistently observed that dispositional gratitude moderated the relationship between SES and interleukin-6 (as a biomarker of inflammation). Consistent with our first hypothesis, we found significant associations between education attainment and income (as indices of SES) and interleukin-6 among individuals with low dispositional gratitude. However, among individuals with high dispositional gratitude, the associations between SES and the inflammation biomarker disappeared. Importantly, the differential patterns of results as a function of dispositional gratitude were still evident when demographics, health status, health behaviours, and personality traits were controlled for. These findings suggest that individuals with high dispositional gratitude are less likely to be affected by the negative effects of socioeconomic disparities on physical health, thus establishing gratitude as a potential psychological resource in dealing with daily life stressors beyond other trait factors such as personality.

One possible reason why the health of individuals with higher dispositional gratitude is less likely to be affected by their socioeconomic circumstances could be due to grateful individuals’ tendency to be content, optimistic, and adopt a healthier outlook and approach to life’s challenges^27,28^. As studies have identified how people appraise stressors as a key factor underlying socioeconomic disparities in health^11,50^, dispositional gratitude likely functions as a psychological resource that essentially fortifies people against daily stressors. Gratitude may also enhance health through shifts in affective perceptions of daily life events from negative to positive, which have been found to correlate with mood and health^51^. Due to its orientation towards positive appraisal, gratitude is also likely to be incompatible with cognitive beliefs associated with depression, specifically negative views about oneself, the world, and the future^17^. Conversely, gratitude has been linked with hedonic wellbeing and eudaimonic wellbeing^18,26^, both of which are associated with reduced likelihood of depression^20,21^ and improved immune function^26^. Thus, those who are especially grateful are more likely to appraise socioeconomic stressors positively and be less psychologically vulnerable when facing difficulties. With a greater willingness to appreciate one’s circumstances including the trials and tribulations of life, grateful individuals are more likely to engage in effective problem-solving behaviours and adaptive coping strategies when dealing with socioeconomic stressors^30,31^.

While we found narrower socioeconomic disparities in inflammation risk among individuals with higher dispositional gratitude, we did not find a main effect of dispositional gratitude on interleukin-6 in all of our models. At first sight, this seems to runs counter to previous studies that suggest potential positive associations between gratitude and health^34^ and also appears to show that dispositional gratitude is unrelated to positive health outcomes. At least a few considerations are worth making before we can concede that our results are indeed inconsistent with the literature. First, very few studies have examined the effects of gratitude on health using inflammatory biomarkers. Among the few that have done so, we note that their results are not particularly unequivocal. For instance, in the study of gratitude journaling versus treatment-as-usual on health outcomes of heart failure patients^30^, the authors cautioned that the generalizability of their interleukin-6 results are limited by the fact that there were systemic differences between the two patient groups examined. Furthermore, although a positive effect of gratitude journaling on health and other wellbeing outcomes was apparently found, their study did not include a condition where patients spend time writing about non-gratitude-related content (i.e., writing control condition). Without this comparison, we cannot rule out the possibility that the positive effects on health were due to the relaxing benefits of writing in general, rather than engagement in a gratitude mindset specifically. Second, our results appear to mirror those of the study on spirituality, gratitude, and wellbeing of heart failure patients^34^, in that the benefits of gratitude on physical health may be especially due to its psychological and motivational aspects (which then promote better lifestyle or coping behaviors leading to better physical health) rather than a direct link with inflammatory biomarkers and physical health. In their study, which examined the gratitude pathway through which spirituality influences wellbeing outcomes, the only outcome that was unrelated to spirituality was that of the physical health biomarkers, whereas the spirituality-gratitude mediation applied for all other psychological and behavioral indicators. This suggests that the beneficial effects of gratitude may be limited to attenuating the negative effects of psychological stress through healthy coping behaviors rather than direct improvements to physical health and, thus, may account for our results as well. More generally, these issues with our findings and the literature are not so much a sign of the ineffectiveness of gratitude but rather an indication that more studies are needed to understand the promising and nuanced effects of gratitude on physical health outcomes.

Although the current study employed a large sample size and was able to rule out a large number of confounding factors including demographics, health status, health behaviours, and personality traits, some limitations exist. The cross-sectional design of the current study necessitates that causal inferences should be derived with caution from the findings. For example, although the significant interactions between gratitude and SES on interleukin-6 may suggest that gratitude reduces socioeconomic disparities in inflammation risk, a third unforeseen variable may also account for the interaction observed in the current study. Therefore, future research, especially experimental studies, are highly warranted to confirm the causal relations between gratitude and socioeconomic disparities in interleukin-6. This is plausible since research has shown that gratitude is malleable and can be subjected to intervention^52,53^. Another notable limitation is that the current study was based solely on U.S. samples which limits the generalizability of our findings to other cultures and the use of shortened version of gratitude scale may restrict the predictive power of the gratitude construct. Thus, it is also important that future studies attempt to replicate our findings with samples from other populations and triangulate our findings with different measures of gratitude.

In summary, although we did not find strong evidence that gratitude is associated with lower levels of interleukin-6 as a biomarker of health among midlife adults in the U.S., in the context of SES, we found some evidence that dispositional gratitude narrowed the negative impact of low SES on health. Specifically, we found socioeconomic disparities in interleukin-6 for individuals with low gratitude but not for individuals with high gratitude. These findings highlight the importance of considering gratitude when examining socioeconomic disparities in inflammation risk. Gratitude may not directly cause health improvements, but being grateful and appreciative of life may cultivate the psychological wherewithal to fortify and buffer oneself from daily stressors through positive appraisals of challenges and the willingness to adopt coping strategies. While firm conclusions from the current study are still premature, the findings suggest that further research on gratitude as a psychological resource can be promising for our understanding of health and the development of effective health interventions.

## Method

### Participants

Participants in the current study consisted of 1,054 adults who completed the Midlife in the United States II: Biomarker Project conducted between 2004–2009^54^. The sample is a subset of a large-scale longitudinal project from the original MIDUS 1 survey that began in 1995, with 7,108 noninstitutionalized adults recruited through random digit sampling from 48 contiguous states. In the Biomarker Project, participants were invited for an overnight hospital stay in one of three general clinical research centers in the United States (University of California, Los Angeles; Georgetown University; and University of Wisconsin-Madison), during which participants underwent a physical exam that included the collection of a fasting blood sample before breakfast on the second day of the participant’s hospital stay^55^. Participants’ demographic, health-related information, and personality characteristics are summarized in Table 3. The data collection was approved by the Health Sciences IRBs at the University of Wisconsin-Madison (H-2008-0060) and was conducted in accordance with the approved guidelines and regulations. All participants provided written informed consent prior to their participation.

**Table 3:** Descriptive Statistics for Demographics, Health Status, Health Behaviours, and Personality Characteristics.

	n	M	SD	Range
Demographic				
Age (years)	1,054	58.04	11.62	35–86
Gender (% of male)	1,054	45.26%		
Marital status (% of married)	1,054	69.83		
Citizenship (US citizen)	1,054	99.6%		
Education^a^	1,051	7.74	2.45	1–12
Income	1,013	45,010	40,462	0–200,000
Income group (quartile)	1,013	2.50	1.12	1–4
Health Status and Behaviours				
Number of chronic disease	1,054	4.02	2.94	0–20
Non-smoker (%)	1,054	55.3%		
Former smoker (%)	1,054	33.3%		
Current smoker (%)	1,054	11.4%		
Alcohol consumption	1,054	2.53	1.58	1–6
Personality^b^				
Openness to experience	1,045	2.96	0.52	1–4
Conscientious	1,050	3.40	0.45	1–4
Extraversion	1,050	3.13	0.57	1–4
Agreeableness	1,050	3.44	0.50	1–4
Neuroticism	1,050	2.03	0.63	1–4

Note. SDs are shown in parentheses.

^a^Education attainment was rated on a scale of 1 (No school) to 12 (Ph.D, ED. D, MD, LLB, LLD, JD, or other professional degree).

^b^Each personality score was calculated by averaging the respective personality items that were rated on a four-point Likert scale (*1* = *not at all*, *4* = *a lot*; Rossi^64^), with higher scores reflecting a higher amount of that particular personality dimension (e.g., greater openness to experience).

### Measures

#### Interleukin-6

Fasting blood samples were collected from each participant before breakfast on the second day of their hospital stay and stored in a −60 °C to −80 °C freezer, which were subsequently shipped in a dry ice container to the MIDUS Biocore laboratory. Serum-level Interleukin-6 (interleukin-6) was measured using the Quantikine® high-sensitivity enzyme-linked immunosorbent assay (ELISA) kit #HS600B (R&D Systems, Minneapolis, MN). The assay range and reference range were 0.156–10 pg/mL and 0.45–9.96 pg/mL respectively. The laboratory inter-assay coefficient of variance was 12.31% and the intra-assay coefficient of variance was 3.25%, which were within an established acceptable range^56^. Higher interleukin-6 values indicate greater inflammatory risk.

#### Socioeconomic status

SES was operationalized by two commonly used and well-established SES indicators—education attainment and income^57^. Education attainment was measured in a telephone interview where participants were asked about the highest level of education they had achieved. Education attainment was rated on a scale of 1 (*No school*) to 12 (*Ph*.*D*, *ED*. *D*, *MD*, *LLB*, *LLD*, *JD*, *or other professional degree*) as a continuous variable. Income was measured based on participants’ total personal earning income, pension income, and social security income. Based on the procedure of past studies^57,58^, we stratified income into quartiles and operationalized them as a continuous variable in our analyses (Q1: less than $16,000; Q2: $17,000-$34,000; Q3: $34,750-$61,250; Q4: more than $62,500).

#### Dispositional gratitude

Dispositional gratitude was measured with a two-item shortened version of the gratitude scale adapted from McCullough, Emmons, and Tsang’s article^59^. Participants rated their agreement with the statements, “I have so much in life to be thankful for” and “I am grateful to a wide variety of people” on a seven-point Likert scale (1 = strongly disagree, 7 = strongly agree). Despite the small number of items, the shortened version of the gratitude scale has acceptable reliability (*α* = 0.721). More importantly, it has demonstrable criterion validity in predicting theoretical relevant constructs such as age, gender, life satisfaction, extraversion, self-esteem, and optimism^24,60–62^.

### Data analysis

The current study aimed to examine the moderating role of dispositional gratitude on the relationship between indicators of SES – including (1) education attainment and (2) income – and interleukin-6. To test our hypotheses, we conducted a series of moderation analyses using the SPSS PROCESS macro^63^ to determine the significance of the interaction between SES and gratitude on interleukin-6. The PROCESS macro employed ordinary least squares regressions to estimate the coefficients of each predictor and their interactions. A significant two-way interaction of SES × gratitude would support our hypothesis that gratitude moderates the relationship between SES and interleukin-6. To minimize multicollinearity, two separate moderation analyses were conducted for education attainment and income as the independent variable. When a significant two-way interaction of SES × gratitude was observed, simple slopes were computed to further probe the interaction effect. Rather than using a specific cut-off, our simple-slope analyses were based on the overall mean and standard deviation of the education attainment and income of our sample.

For each SES indicator, we examined four separate models each with an additional set of covariates to ensure the robustness of the hypothesized moderation. In the first model, we presented an unadjusted model with SES (either education attainment or income), gratitude, and the SES × gratitude interaction as the only predictors without including any covariates. In the second model, we entered participants’ demographics, including age, gender, marital status, and citizenship, as the covariates. In the third model, we further controlled for the total number of chronic diseases experienced in the past 12 months as an indicator of health status and health-impacting behaviours such as smoking and alcohol consumption. In the fourth model, we controlled for openness to experience, conscientiousness, extraversion, agreeableness, and neuroticism to ensure that our hypothesized pattern was not an artefact of personality^64^. In each model, interleukin-6 and dispositional gratitude were log-transformed to reduce skew in the distributions. Interleukin-6 was also winsorized to 4 standard deviations from the mean to minimize the influence of extreme outliers (*n* = 14). Missing values (less than 4% on any given variable) were imputed using the expectation-maximization (EM) algorithm^65^.

## Data Availability

Data and materials from the MIDUS II: Biomarker Project are available from the Inter-University Consortium for Political and Social Research (http://www.icpsr.umich.edu).
